# The association of dietary macronutrients with anthropometric changes, using iso-energetic substitution models: Tehran lipid and glucose study

**DOI:** 10.1186/s12986-019-0411-2

**Published:** 2019-11-27

**Authors:** Firoozeh Hosseini-Esfahani, Glareh Koochakpoor, Parvin Mirmiran, Samira Ebrahimof, Fereidoun Azizi

**Affiliations:** 1 Nutrition and Endocrine Research Center, Research Institute for Endocrine Sciences, Shahid Beheshti University of Medical Sciences, Tehran, Iran; 2 Maragheh University of Medical Sciences, Maragheh, Iran; 3 Endocrine Research Center, Research Institute for Endocrine Sciences, Shahid Beheshti University of Medical Sciences, Tehran, Iran

**Keywords:** Macronutrients composition, Plant proteins, Replacement, Obesity

## Abstract

**Background:**

The consequences of optimal dietary macronutrient compositions especially quality of proteins on weight gain still remain controversial. The aim of the current study was to evaluate the iso-energetic substitution of dietary macronutrients in relation to anthropometric changes.

**Methods:**

This prospective study was conducted on 2999 men and 4001 women aged 20–70 years who were followed for 3.6 years. A valid and reliable 168-item semi-quantitative food frequency questionnaire was used to assess usual dietary intakes. Weight (kg) and waist circumference (WC) (cm) changes were calculated by subtracting the weight and WC at baseline from their measurements at follow up. Participants were divided into two groups; those with no change or decrease in weight or WC and those with increase in weight or WC. Dietary macronutrients (percentage of energy) divided by 5 to calculate one unit.

**Results:**

A one unit higher proportion of carbohydrates at the expense of all types of fatty acids was associated with weight loss in men (*P* < 0.05). A one unit higher proportion of plant proteins at the expense of animal protein (β = − 0.84), non-starch carbohydrates (β = − 0.86), saturated fat (β = − 0.76), mono-unsaturated fat (β = − 0.76) and poly-unsaturated fat (β = − 0.86) was associated with weight loss (*P* < 0.05). A one unit higher proportion of plant proteins at the expense of animal proteins (OR: 0.49), non-starch carbohydrates (OR: 0.49), saturated fat (OR: 0.49), mono-unsaturated fat (OR: 0.49), and poly-unsaturated fat (OR: 0.48) was associated with a lower risk of increase in WC (*P* < 0.05).

**Conclusions:**

A higher proportion of dietary plant protein in replacement of simple carbohydrates, fats and animal proteins was associated with a lower increase in weight or WC.

## Introduction

Obesity is a growing epidemic of the twenty-first century in developed and developing countries [1]. It is associated with an increased risk of serious health problems including cardiovascular diseases, diabetes and several cancers [2]. The appropriate method for its prevention and treatment is widely acknowledged. Weight gain often occurs gradually over a lifetime due to imbalance of energy intake and expenditure [3]. In addition, macronutrient composition has also been suggested as an important determinant in the obesity epidemic [4]; carbohydrate, fat and protein intake are simultaneously considered as macronutrient balance or overall diet, and in an iso-energetic setting the differences in one dietary macronutrient reflect substitutions for other macronutrients. Analyzing the association between macronutrient intakes and weight change in statistical methods which account that one macronutrient is substituted with the other, may help clarify the complex relationships. Several previous randomized clinical trials [5–7] have shown that increase in protein percentage in iso-caloric diets had advantages in terms of adiposity, blood lipid profiles, cardiovascular risk markers and insulin levels; however, in short term controlled trials, high protein, moderate or low carbohydrate diets result in more weight loss than traditional low fat, high carbohydrate diets [7]. Long term intervention studies indicate equivalent weight loss with the low fat (< 30% of energy from fat) and low carbohydrate (< 50% of energy from carbohydrate) approaches [6]. Another study on healthy individuals showed that higher protein intake was associated with weight gain [8]. Moreover, one study showed differences in animal and plant protein, with a direct association of animal protein with the risk of overweight and obesity, and an inverse association for plant protein [9]. Therefore, the consequences of optimal macronutrient composition of the usual diet still remain controversial in observational studies. In addition, the debate on protein sources is ongoing, addressing the nutritional quality of dietary proteins and their amino acid composition. Hence, the aim of the current study was to evaluate the substitution dietary macronutrient intakes with each other in relation to anthropometric changes in a group of Tehranian adults.

## Materials and methods

### Study population

This study was performed within the framework of the Tehran Lipid and Glucose Study (TLGS), an ongoing longitudinal community-based study originated in 1999 with the aim of determining the prevalence of risk factors of non-communicable diseases in a representative sample of the urban population of Tehran, capital of Iran [10]. The first survey was a cross-sectional study (1999–2001) on 15,005 subjects aged ≥3 years, and follow up examinations have been conducted every 3 years (2002–2005, 2005–2008, 2009–2011 and 2012–2015) to identify new developed diseases or risk factors. Details of this ongoing cohort study have been published elsewhere [11]. Of 12,823 individuals who entered the 2009–2011 survey of TLGS (baseline of our study), 7344 subjects aged 20–70 years were randomly selected for dietary assessment, based on age- and sex-stratified random sampling and followed until 2012–15 survey of TLGS (end of our study). Individuals who had not completed anthropometrical data at the baseline or follow up survey, those who over- or under-reported and subjects who had remarkable weight or waist circumference (WC) change (±5SD) were excluded (*n* = 344). To define over- and under-reports, the estimated energy intake (EI) was divided by the estimated energy requirement (EER); subjects were excluded when EI:EER was not within the ±3SD of mean value. Data of 7000 participants (2999 men and 4001 women) were finally available for analysis over a mean 3.6 years of follow up.

### Measurements

Trained interviewers collected demographic data using the pre-tested questionnaire and face to face private interview. A valid and reliable 168-item semi-quantitative food frequency questionnaire (FFQ) was used to assess usual dietary intakes [12, 13]. The consumption frequency of each food item on a daily, weekly, or monthly basis was converted to daily intakes, and portion sizes were then converted to grams using measuring cups and spoons. The Iranian food composition table (FCT) is incomplete; therefore, we used the United States Department of Agriculture (USDA) FCT to analyze foods [14]. However, the Iranian FCT was used for some national foods and beverages when these foods were not listed in the USDA FCT [15]. Macronutrients including carbohydrate, fat, protein, and their subtypes; i.e. saturated fatty acids (SFAs), monounsaturated fatty acids (MUFAs), polyunsaturated fatty acids (PUFAs), starch and non-starch carbohydrates, animal and plant proteins, were the exposures of the current study. Legumes, nuts and vegetables are sources of plant protein, while dairy, meat, poultry and fish are sources of animal protein.

The body weight of each participant was measured to the nearest 100 g using digital scales (Seca, Hamburg, Germany) while subjects were minimally clothed and not wearing shoes. Height was measured to the nearest 0.5 cm using a stadiometer while the subjects were in standing position, with their shoulders in normal alignment and without shoes. WC was taken at the end of a normal expiration, over light clothing, using a flexible and non-stretched measurement tape positioned at the level of umbilicus, without exerting any pressure on the body surface; measurements were recorded to the nearest 0.1 cm. All measurements were carried out by one examiner for women and one for men to avoid subjective errors. Detailed measurements of variables in the TLGS have been reported elsewhere [10]. Weight (kg) and WC (cm) changes were calculated by subtracting the weight and WC at baseline from their measurements at follow up; increase in weight and WC was defined if weight or WC changes were positive or > 0.

Physical activity was assessed in Tehranian adults using the Persian-translated modifiable activity questionnaire (MAQ) [16, 17] with high reliability and moderate validity [18]. Data were gathered on the frequency and time spent on light, moderate, hard and very hard intensity activities according to the list of common activities of daily life over the past year. Physical activity levels were transformed into metabolic equivalent-hours/week (MET/h/week) [16, 18]. The metabolic equivalent values were categorized based on intensity, using guidelines of the American College of Sports Medicine/Centers for Disease Control and Prevention [19].

### Statistical analysis

Statistical analyses were performed using the Statistical Package for Social Sciences (version 20.0; SPSS) (IBM, Armonk, New York, USA). Baseline characteristics of both genders, based on tertiles of percentage of energy from carbohydrate, protein and fat were computed using the ANOVA test for continuous variables as mean ± SD and chi-square test for categorical variables. Dietary macronutrients and their subtypes were adjusted for energy intake using the nutrient density method (percentage of energy) and then divided by 5 to calculate one unit of percentages of energy from macronutrients. Multivariate linear regression analyses were used to investigate the substitution model; these models estimated changes in weight and WC by iso-caloric increase in each macronutrient intake (per one unit increase in percentage of energy intake) as a continuous variable in exchange of 5 unit of percentage of energy from another macronutrient. The coefficients (β) in these models can be explained as estimated anthropometric change by one unit increase in one nutrient at the expense of another not included in the model, while keeping energy intake and other nutrients which are included in the model constant. Also, in these analyses the following demographic and lifestyle covariates were included in models: baseline age (continuous), sex (except sex strata), physical activity (low, moderate and high), smoking (former and current smoker/never smoked), education level (> 12 and ≤ 12 years of education), baseline BMI (in models estimating changes in weight) and baseline WC (in models estimating changes of WC) and energy intake.

Multivariate adjusted logistic regression analyses were used to estimate the odds ratios (95% CIs) of increase in weight or WC in an iso-energetic substitution model; replacement of one unit of energy from one macronutrient at the expense of another macronutrient. All models were adjusted for the above-mentioned demographic and lifestyle covariates, and total energy intake.

Changes in weight and waist were considered significant, when a two tailed *P* value was < 0.05.

## Results

Characteristics of the population based on sex and percentage of energy from each macronutrient are presented in Table 1. Compared to subjects in the first tertile of energy from carbohydrates, subjects in the third tertile were older, had a lower frequency of current smokers, and lower change in weight and WC during follow-up. Men in the third tertile of energy from carbohydrate had a higher frequency of participants with university degrees, compared to the first tertile; while women in the mentioned tertile, this frequency was lower, compared to the first tertile. Compared to subjects in the first tertile of energy from protein, subjects in the third tertile had a higher education level, higher BMI and WC at baseline and reported a lower energy intake. Women in the third tertile of energy from protein were older compared to the first tertile. Compared to subjects in the first tertile of energy from fat, subjects in the third tertile were younger, had a higher weight change during follow-up and reported higher energy intake; they also had a higher percentage of smokers. In men, fewer subjects in the third tertile of energy from fat had university degrees, compared to the first tertile. Also, WC change increased according to the tertiles of energy from fat in women.

**Table 1:** Characteristics of the population according to percentage of energy from carbohydrate, protein and fat by sex: Tehran Lipid and Glucose Study

	Tertiles of percentage of energy from carbohydrate			Tertiles of percentage of energy from protein			Tertiles of percentage of energy from fat		
	1	2	3	1	2	3	1	2	3
Men (*n* = 2999)									
Carbohydrate (% of energy)	52.6 ± 3.5	59.0 ± 1.4	65.2 ± 3.6*	60.8 ± 6.5	60.4 ± 5.3	59.0 ± 5.4*	64.5 ± 3.9	58.9 ± 2.9	53.3 ± 4.9*
Protein (% of energy)	14.9 ± 2.7	14.8 ± 1.9	14.4 ± 2.5*	12.4 ± 1.0	14.4 ± 0.5	17.1 ± 2.3*	14.8 ± 2.1	14.8 ± 2.4	14.1 ± 2.9*
Fat (% of energy)	34.9 ± 4.2	29.2 ± 2.3	24.3 ± 3.4*	29.6 ± 6.2	28.2 ± 4.8	27.9 ± 4.4*	23.9 ± 2.7	29.5 ± 1.3	35.8 ± 3.6*
Age (years)	39.5 ± 13.6	40.6 ± 13.5	43.6 ± 12.9*	41.1 ± 13.0	41.3 ± 13.4	42.5 ± 13.7	43.0 ± 13.0	40.7 ± 13.5	40.4 ± 13.7*
Baseline BMI (kg/m2)	26.7 ± 4.3	26.6 ± 4.2	26.8 ± 4.1	26.4 ± 4.2	26.7 ± 4.1	27.1 ± 4.2*	26.8 ± 4.1	26.7 ± 4.2	26.5 ± 4.2
Weight change (kg)	3.2 ± 7.2	3.1 ± 6.1	2.1 ± 5.7*	2.9 ± 6.1	2.9 ± 6.2	2.5 ± 6.4	2.1 ± 5.5	3.2 ± 6.3	3.0 ± 7.1*
Baseline WC (cm)	94.4 ± 11.5	94.2 ± 10.8	95 ± 10.7	93.7 ± 11.4	94.6 ± 10.7	95.3 ± 10.6*	94.8 ± 10.6	94.5 ± 10.9	94.2 ± 11.5
WC change (cm)	2.8 ± 6.9	2.6 ± 5.6	2.0 ± 5.3*	2.6 ± 5.7	2.4 ± 5.7	2.2 ± 6.2	2.1 ± 5.3	2.6 ± 5.9	2.6 ± 6.7
Energy intake (kcal)	2545 ± 727	2476 ± 650	2443 ± 680	2572 ± 685	2445 ± 667	2426 ± 686*	2424 ± 678	2492 ± 658	2564 ± 719*
Educational level (> 12 years)	23.1	29	27.1*	21.7	28.2	30.7*	30.6	25.8	21.8*
Physical activity status (%)									
Sedentary	75.9	80.5	81.6	81.0	79.3	79.1	81.5	79.6	77.1
Moderate	13.7	12.3	11.0	10.9	12.1	13.4	11.2	12.9	12.5
Active	10.4	7.2	7.4	8.1	8.6	7.5	7.3	7.5	10.4
Former/Current smoker (%)	44.1	37.3	37.3*	42.3	37.0	37.8	36.2	37.4	53.7*
Women (*n* = 4001)									
Carbohydrate (% of energy)	52.0 ± 3.8	58.8 ± 1.4	65.2 ± 4.2*	57.5 ± 7.2	58.4 ± 5.5	57.5 ± 6.0*	64.6 ± 4.3	58.7 ± 2.9	52.6 ± 4.9*
Protein (% of energy)	14.6 ± 2.6	14.8 ± 3.5	14.4 ± 3.0	12.2 ± 1.1	14.4 ± 0.5	17.3 ± 3.5*	15.3 ± 3.9	15.0 ± 2.2	14.0 ± 2.8*
Fat (% of energy)	36.2 ± 4.6	29.9 ± 2.6	25.0 ± 3.3*	33.3 ± 6.9	30.4 ± 5.1	29.7 ± 4.9*	24.2 ± 2.4	29.7 ± 1.3	36.7 ± 4.2*
Age (years)	38.3 ± 12.2	39.7 ± 12.5	42.2 ± 13.1*	39.1 ± 12.5	38.5 ± 12.3	41.8 ± 12.9*	43.1 ± 13.4	39.3 ± 12.2	38.3 ± 12.2*
Baseline BMI (kg/m^2^)	27.3 ± 5.2	27.2 ± 4.9	27.1 ± 5.2	27.1 ± 5.2	27.3 ± 5.1	28.1 ± 5.1*	28.0 ± 5.1	27.5 ± 5.1	27.2 ± 5.2*
Weight change (kg)	2.6 ± 5.8	2.4 ± 5.6	2.0 ± 5.4*	2.5 ± 5.4	2.5 ± 5.9	2.2 ± 5.6	1.7 ± 5.4	2.5 ± 5.6	2.7 ± 5.8*
Baseline WC (cm)	85.6 ± 13.5	85.4 ± 12.7	85.2 ± 13.4	85.5 ± 13.4	85.6 ± 13.3	87.5 ± 13.0*	88.2 ± 13.2	85.9 ± 13.1	85.2 ± 13.3*
WC change (cm)	6.1 ± 8.1	6.2 ± 8.0	5.2 ± 8.0*	6.1 ± 8.0	5.8 ± 8.2	5.8 ± 8.0	5.1 ± 7.8	6.3 ± 8.2	6.1 ± 8.1*
Energy intake (kcal)	2276 ± 661	2245 ± 632	2234 ± 679*	2319 ± 674	2268 ± 647	2187 ± 643*	2192 ± 672	2261 ± 633	2297 ± 663*
Educational level (> 12 years)	20.3	20.5	16.8*	15.5	22.2	20.8*	17.1	21.4	19.3*
Physical activity status (%)									
Sedentary	84.7	84.5	85.9	85.3	85.2	84.4	86.7	84.8	84.0
Moderate	11.6	12.0	10.7	11.3	10.9	12.1	9.9	11.9	12.1
Active	3.7	3.5	3.4	3.4	3.9	3.6	3.4	3.3	3.9
Smoking (%)									
Former/Current smoker	5.1	4.8	2.8*	5.4	3.8	3.8	2.5	4.4	5.6*

*BMI* Body mass index, *WC* Waist circumference, Weight (kg) and WC (cm) change were calculated by subtracting the weight and WC at baseline from their measurements at follow up; increase in weight and WC were defined if weight or WC change were positive or > 0

The metabolic equivalent values were categorized according to intensity using guidelines of the American College of Sports Medicine/Centers for Disease Control and Prevention (ACSM/CDC). *P < 0.05

Adjusted weight change for the iso-energetic replacement of 5% of energy from one macronutrient by another macronutrient is presented in Table 2. A 1 unit higher proportion of plant protein at the expense of animal protein (β = − 0.84), non-starch carbohydrates (β = − 0.86), saturated fat (β = − 0.76), mono-unsaturated fat (β = − 0.76), and poly-unsaturated fat (β = − 0.86) was associated with weight loss (*P* < 0.05). A 1 unit higher proportion of plant protein at the expense of animal protein, non-starch carbohydrates and saturated and poly-unsaturated fatty acids was associated with decreased risk of weight attainment (Table 3). The substitution of other macronutrients was not consistently associated with weight change.

**Table 2:** Adjusted weight change for iso-energetic replacement of macronutrients

Type of macronutrient Substitution	All		Men		Women	
	β	*P*-value	β	*P*-value	β	*P*-value
Carbohydrate ↑						
Fat ↓	0.00	0.96	−0.03	0.04	0.01	0.65
Protein ↓	−0.01	0.57	−0.04	0.25	0.01	0.71
Carbohydrate ↑						
SFA ↓	−0.01	0.47	−0.06	0.02	0.01	0.65
MUFA ↓	−0.03	0.11	−0.06	0.02	−0.00	0.90
PUFA ↓	−0.02	0.24	−0.05	0.03	0.01	0.62
Protein ↓	−0.03	0.10	− 0.03	0.14	− 0.00	0.92
Carbohydrate ↑						
Fat ↓	− 0.01	0.68	− 0.02	0.33	0.01	0.52
Plant Protein ↓	−0.02	0.47	−0.03	0.22	0.01	0.72
Animal Protein ↓	−0.02	0.46	−0.04	0.25	0.01	0.73
Carbohydrate ↑						
SFA ↓	−0.01	0.56	−0.02	0.47	0.01	0.68
MUFA ↓	−0.03	0.14	−0.05	0.07	−0.00	0.95
PUFA ↓	−0.02	0.33	−0.04	0.11	0.01	0.51
Plant Protein ↓	−0.03	0.10	−0.06	0. 17	−0.00	0.92
Animal Protein ↓	−0.03	0.01	−0.04	0. 86	−0.00	0.92
Plant protein ↑						
Animal protein ↓	0.01	0.46	−0.04	0.04	0.01	0.46
Carbohydrate ↓	−0.08	0.37	−0.04	0.03	−0.01	0.69
Fat ↓	−0.07	0.43	−0.04	0.04	−0.09	0.62
Plant protein ↑						
Animal protein ↓	−0.69	0.05	−0.08	0.01	0.03	0.96
Starch ↓	−0.17	0.12	−0.05	0.03	−0.18	0.39
Non-starch ↓	−0.72	0.04	−0.08	0.00	−0.00	0.10
Fat ↓	−0.71	0.04	−0.08	0.01	0.03	0.96
Plant protein ↑						
Animal protein ↓	0.01	0.52	−0.05	0.02	0.01	0.57
Carbohydrate ↓	−0.11	0.23	−0.06	0.00	−0.05	0.77
SFA ↓	−0.06	0.52	−0.04	0.04	−0.03	0.86
MUFA ↓	−0.08	0.37	−0.05	0.02	−0.06	0.74
PUFA ↓	−0.09	0.30	−0.05	0.01	−0.11	0.54
Plant protein ↑						
Animal protein ↓	−0.84	0.02	−0.11	0.00	0.14	0.84
Starch ↓	−0.20	0.07	−0.06	0.00	−0.15	0.49
Non-starch ↓	−0.86	0.02	−0.11	0.00	0.11	0.87
SFA ↓	−0.76	0.03	−0.10	0.00	0.17	0.79
MUFA ↓	−0.76	0.03	−0.093	0.01	0.06	0.93
PUFA ↓	−0.86	0.02	−0.11	0.00	−0.00	0.99

*SFA* Saturated fatty acid, *MUFA* Mono-unsaturated fatty acids, *PUFA* Poly-unsaturated fatty acids. Non-starch carbohydrates: simple sugars. The percentage of energy from macronutrients divides by 5 to calculate one unit of each macronutrient. Linear regression analysis was used to estimate weight change for the iso-energetic increase (↑) of 1unit of percentage of energy from one macronutrient at the expense of another (↓). Models were adjusted for baseline age (continuous), sex (except sex strata), physical activity (low, moderate and high), smoking (former and current smoker/never smoked), education le vel (> 12 and ≤ 12 years of education), baseline BMI and energy intake

**Table 3:** Odds ratio of increase in weight according to iso-energetic substitution of dietary macronutrients

Type of macronutrient Substitution	All		Men		Women	
	OR	95% CI	OR	95% CI	OR	95% CI
Carbohydrate ↑						
Fat ↓	0.96	0.92–1.00	0.98	0.91–1.05	0.94	0.89–1.00^*^
Protein ↓	0.94	0.86–1.02	0.92	0.79–1.05	0.96	0.85–1.07
Carbohydrate ↑						
SFA ↓	0.94	0.89–0.99	0.96	0.88–1.04	0.92	0.86–0.99
MUFA ↓	0.91	0.85–0.97	0.90	0.82–0.10	0.91	0.84–0.99
PUFA ↓	0.98	0.89–0.99	0.94	0.86–1.02	0.94	0.87–1.01
Protein ↓	0.91	0.85–0.97	0.91	0.83–1.01	0.91	0.84–0.99
Carbohydrate ↑						
Fat ↓	0.96	0.92–1.01	0.99	0.92–1.07	0.96	0.90–1.02
Plant Protein ↓	0.94	0.86–1.02	0.89	0.77–1.03	0.96	0.85–1.07
Animal Protein ↓	0.94	0.86–1.02	0.91	0.79–1.05	0.95	0.85–1.07
Carbohydrate ↑						
SFA ↓	0.94	0.89–0.10	0.99	0.90–1.08	0.93	0.86–1.01
MUFA ↓	0.91	0.85–0.97	0.93	0.84–1.03	0.92	0.84–1.00
PUFA ↓	0.97	0.89–0.10	0.99	0.87–1.05	0.95	0.88–1.02
Plant Protein ↓	0.91	0.85–0.97	0.91	0.82–1.01	0.91	0.84–0.99
Animal Protein ↓	0.91	0.85–0.97	0.93	0.84–1.04	0.91	0.84–0.99
Plant protein ↑						
Animal protein ↓	1.01	0.99–1.02	0.81	0.63–0.98	1.01	0.98–1.03
Carbohydrate ↓	0.97	0.90–1.05	0.79	0.61–0.95	0.93	0.84–1.03
Fat ↓	0.98	0.91–1.06	0.82	0.64–0.97	0.93	0.84–1.04
Plant protein ↑						
Animal protein ↓	0.76	0.57–0.10	0.68	0.44–1.06	0.85	0.58–1.23
Starch ↓	0.96	0.87–1.05	0.76	0.57–0.10	0.94	0.83–1.06
Non-starch ↓	0.75	0.57–0.10	0.65	0.42–1.02	0.87	0.61–1.29
Fat ↓	0.75	0.57–0.10	0.65	0.42–1.01	0.87	0.60–1.27
Plant protein ↑						
Animal protein ↓	1.00	0.99–1.02	0.79	0.61–0.98	1.01	0.98–1.03
Carbohydrate ↓	0.96	0.89–1.03	0.74	0.57–0.85	0.92	0.83–1.03
SFA ↓	0.99	0.92–1.07	0.82	0.63–0.99	0.96	0.86–1.07
MUFA ↓	0.98	0.91–1.06	0.79	0.61–1.02	0.95	0.85–1.06
PUFA ↓	0.97	0.90–1.05	0.76	0.59–0.98	0.93	0.84–1.04
Plant protein ↑						
Animal protein ↓	0.72	0.54–0.96	0.60	0.38–0.95	0.87	0.59–1.27
Starch ↓	0.95	0.86–1.04	0.71	0.53–0.93	0.94	0.83–1.07
Non-starch ↓	0.73	0.55–0.97	0.55	0.35–0.88	0.90	0.62–1.32
SFA ↓	0.74	0.56–0.99	0.60	0.38–0.95	0.90	0.61–1.31
MUFA ↓	0.75	0.57–1.00	0.63	0.40–0.99	0.89	0.61–1.30
PUFA ↓	0.72	0.54–0.96	0.56	0.35–0.89	0.88	0.60–1.28

*OR* Odds ratio, *CI* Confidence interval, *SFA* Saturated fatty acid, *MUFA* Mono-unsaturated fatty acids, *PUFA* Poly-unsaturated fatty acids, Non-starch carbohydrates: simple sugars

Weight (kg) change was calculated by subtracting the weight at baseline from their measurements at follow up; increase in weight was defined if weight was positive or > 0. The percentage of energy from macronutrients divides by 5 to calculate one unit of each macronutrient. Logistic regression analysis was applied to estimate OR of weight change for the iso-energetic increase (↑) of 1 unit of percentage of energy from one macronutrient at the expense of another (↓). Models were adjusted for baseline age (continuous), sex (except sex strata), physical activity (low, moderate and high), smoking (former and current smoker/never smoked), education level (> 12 and ≤ 12 years of education), baseline BMI and energy intake

A 5 unit higher proportion of plant protein at the expense of animal protein (β = − 1.60), non-starch carbohydrates (β = − 1.60), saturated fat (β = − 1.54), mono-unsaturated fat (β = − 1.57), and poly-unsaturated fat (β = − 1.61) was associated with WC loss (*P* < 0.05) (Table 4).

**Table 4:** Adjusted waist circumference change for iso-energetic replacement of macronutrients

Type of macronutrient Substitution	All		Men		Women	
	β	*P*-value	β	*P*-value	β	*P*-value
Carbohydrate ↑						
Fat ↓	−0.01	0.49	−0.05	0.80	−0.01	0.68
Protein ↓	−0.01	0.53	−0.03	0.45	0.00	0.93
Carbohydrate ↑						
SFA ↓	− 0.01	0.43	− 0.03	0.24	0.01	0.73
MUFA ↓	− 0.02	0.33	− 0.04	0.08	0.01	0.75
PUFA ↓	−0.01	0.534	−0.02	0.29	0.01	0.65
Protein ↓	−0.02	0.30	−0.04	0.08	0.01	0.74
Carbohydrate ↑						
Fat ↓	0.00	0.75	0.01	0.62	0.00	0.88
Plant Protein ↓	−0.01	0.58	−0.03	0.38	0.00	0.84
Animal Protein ↓	−0.01	0.53	−0.03	0.46	0.00	0.94
Carbohydrate ↑						
SFA ↓	0.00	0.75	−0.00	0.99	0.02	0.43
MUFA ↓	0.00	0835	−0.02	0.37	0.02	0.50
PUFA ↓	0.00	0.91	−0.01	0.78	0.01	0.47
Plant Protein ↓	0.02	0.31	−0.04	0.07	0.01	0.69
Animal Protein ↓	0.00	0.87	−0.02	0.41	0.01	0.50
Plant protein ↑						
Animal protein ↓	0.01	0.59	−0.05	0.00	0.01	0.54
Carbohydrate ↓	−0.12	0.14	−0.05	0.00	−0.15	0.48
Fat ↓	−0.12	0.16	−0.05	0.01	−0.17	0.31
Plant protein ↑						
Animal protein ↓	−1.01	0.00	−0.11	0.00	−0.46	0.43
Starch ↓	−0.17	0.11	−0.07	0.00	−0.15	0.44
Non-starch ↓	−0.10	0.00	−0.12	0.00	−0.41	0.49
Fat ↓	−0.10	0.00	−0.12	0.00	−0.40	0.50
Plant protein ↑						
Animal protein ↓	−0.05	0.00	−0.01	0.00	−0.03	0.07
Carbohydrate ↓	−0.05	0.00	−0.07	0.00	−0.03	0.08
SFA ↓	−0.05	0.00	−0.03	0.68	−0.06	0.00
MUFA ↓	−0.05	0.00	−0.06	0.00	−0.03	0.05
PUFA ↓	−0.05	0.00	−0.07	0.00	−0.03	0.07
Plant protein ↑						
Animal protein ↓	−1.60	0.00	−0.14	0.00	−1.11	0.08
Starch ↓	−0.11	0.32	−0.09	0.00	−0.11	0.57
Non-starch ↓	−1.60	0.00	−0.15	0.00	−1.11	0.48
SFA ↓	−1.54	0.00	−0.14	0.00	−1.11	0.08
MUFA ↓	−1.57	0.00	−0.13	0.00	−1.11	0.08
PUFA ↓	−1.61	0.00	−0.15	0.00	−1.15	0.08

*SFA* Saturated fatty acid, *MUFA* Mono-unsaturated fatty acids, *PUFA* Poly-unsaturated fatty acids. Non-starch carbohydrates: simple sugars

The percentage of energy from macronutrients divides by 5 to calculate one unit of each macronutrient. Estimating waist circumference change was calculated using linear regression analysis for the iso-energetic increase (↑) of 1 unit of percentage of energy from one macronutrient at the expense of another (↓). Models were adjusted for baseline age (continuous), sex (except sex strata), physical activity (low, moderate and high), smoking (former and current smoker/never smoked), education level (> 12 and ≤ 12 years of education), baseline waist circumference and energy intake

Odds ratio (OR) of increase in WC according to an iso-energetic substitution of macronutrients is shown in Table 5. A 1 unit higher proportion of plant protein at the expense of animal protein, non-starch carbohydrates or fatty acids was associated with a lower risk of WC increase in men and women (Table 5).

**Table 5:** Odds ratio of increase in waist circumference according to iso-energetic substitution of dietary macronutrients

Type of macronutrient Substitution	All		Men		Women	
	OR	95% CI	OR	95% CI	OR	95% CI
Carbohydrate ↑						
Fat ↓	1.01	0.96–1.06	0.10	0.93–1.07	1.02	0.95–1.09
Protein ↓	1.01	0.92–1.11	0.94	0.82–1.09	1.11	0.96–1.28
Carbohydrate ↑						
SFA ↓	0.98	0.93–1.04	0.95	0.88–1.04	1.02	0.94–1.11
MUFA ↓	0.97	0.91–1.03	0.91	0.83–1.09	1.04	0.94–1.15
PUFA ↓	0.99	0.93–1.05	0.95	0.87–1.03	1.05	0.96–1.15
Protein ↓	0.97	0.91–1.04	0.92	0.84–1.02	1.04	0.94–1.15
Carbohydrate ↑						
Fat ↓	1.02	0.97–1.07	1.02	0.95–1.11	1.04	0.96–1.11
Plant Protein ↓	1.01	0.92–1.11	0.92	0.79–1.07	1.11	0.95–1.29
Animal Protein ↓	1.01	0.92–1.11	0.94	0.82–1.09	1.10	0.98–1.27
Carbohydrate ↑						
SFA ↓	0.99	0.93–1.05	0.98	0.89–1.07	1.06	0.96–1.16
MUFA ↓	0.98	0.91–1.04	0.95	0.85–1.05	1.05	0.95–1.17
PUFA ↓	0.99	0.94–1.06	0.98	0.89–1.07	1.06	0.96–1.18
Plant Protein ↓	0.97	0.91–1.04	0.92	0.83–1.01	1.04	0.94–1.15
Animal Protein ↓	0.97	0.91–1.04	0.95	0.86–1.06	1.04	0.94–1.15
Plant protein ↑						
Animal protein ↓	1.00	0.99–1.02	0.65	0.50–0.85	1.00	0.98–1.03
Carbohydrate ↓	0.97	0.89–1.04	0.63	0.48–0.83	0.95	0.84–1.07
Fat ↓	0.96	0.89–1.04	0.66	0.51–0.86	0.93	0.82–1.06
Plant protein ↑						
Animal protein ↓	0.49	0.36–0.67	0.40	0.25–0.65	0.63	0.41–0.97
Starch ↓	0.95	0.86–1.04	0.48	0.34–0.68	0.93	0.81–1.08
Non-starch ↓	0.49	0.36–0.67	0.39	0.24–0.63	0.63	0.41–0.97
Fat ↓	0.49	0.36–0.66	0.38	0.23–0.61	0.63	0.41–0.97
Plant protein ↑						
Animal protein ↓	1.00	0.99–1.02	0.66	0.50–0.87	1.01	0.98–1.03
Carbohydrate ↓	0.96	0.89–1.04	0.63	0.48–0.82	0.96	0.84–1.09
SFA ↓	0.97	0.89–1.05	0.68	0.52–0.89	0.93	0.82–1.07
MUFA ↓	0.97	0.89–1.04	0.64	0.49–0.85	0.94	0.82–1.07
PUFA ↓	0.96	0.89–1.04	0.64	0.48–0.84	0.94	0.82–1.07
Plant protein ↑						
Animal protein ↓	0.491	0.36–0.66	0.39	0.24–0.62	0.64	0.42–0.99
Starch ↓	0.947	0.86–1.05	0.50	0.36–0.69	0.95	0.81–1.01
Non-starch ↓	0.489	0.36–0.67	0.38	0.23–0.61	0.64	0.42–0.99
SFA ↓	0.495	0.36–0.68	0.39	0.24–0.63	0.64	0.41–0.99
MUFA ↓	0.487	0.36–0.68	0.37	0.23–0.60	0.64	0.42–0.99
PUFA ↓	0.481	0.35–0.66	0.36	0.22–0.59	0.64	0.41–0.98

*OR* Odds ratio, *CI* Confidence interval, *SFA* Saturated fatty acid, *MUFA* Mono-unsaturated fatty acids, *PUFA* Poly-unsaturated fatty acids. Non-starch carbohydrates: simple sugars. Waist circumference (wc) change was calculated by subtracting the waist at baseline from measurements at follow up; increase in WC was defined if WC was positive or > 0. The percentage of energy from macronutrients divides by 5 to calculate one unit of each macronutrient. Logistic regression analysis was applied for the iso-energetic increase (↑) of 5 unit of percentage of energy from one macronutrient at the expense of another (↓). Models were adjusted for baseline age (continuous), sex (except sex strata), physical activity (low, moderate and high), smoking (former and current smoker/never smoked), education level (> 12 and ≤ 12 years of education), baseline WC and energy intake

## Discussion

In our study, the iso-caloric substitution of carbohydrate with protein was not associated with weight change, whereas the substitution of animal protein, carbohydrate and fatty acids with plant protein was associated with weight loss and reduced WC. This finding can partly justify the contradiction between studies regarding the effect of protein intake on obesity, it seems that the type of protein)animal versus plant protein) is more effective in weight gain than its amount. These results are similar to the findings of another longitudinal study which reported an increase in the risk of obesity with higher animal protein intake, during a 7-year follow up [20]. In contradiction with our findings, two cross sectional studies proposed the use of more animal protein as an effective way for weight loss [21, 22]. Differences in the study design can partly justify this contradiction. Differences in quality, quantity and type of animal protein consumed in different cultures could be another justification for this controversy over the effect of animal protein intake on obesity indices.

Several mechanisms have been proposed for the beneficial effect of plant protein intake on weight control. First, high intake of fiber from plant protein may decrease digestibility, thus, leading to weight loss [23]. Second, plant protein intake increases lean body mass and prevents sarcopenia, leading to an increase in both basal metabolism and physical activity, which in turn reduces the risk of obesity [24]; finally, consumption of legumes increases the expression of proteins involved in fatty acid oxidation, which induce weight loss [25].

In our study, the iso-caloric substitution of fat with carbohydrate decreased the risk of obesity in men. No studies have examined the effects of fat substitution with carbohydrate on obesity, separately in men and women; although, contrary to our results, in two clinical trials, obese subjects lost more weight during 6 months of a carbohydrate-restricted diet than a fat-restricted diet [5, 26]. Diets high in carbohydrate are particularly fattening because of their propensity to elevate insulin secretion, thereby directing fat toward storage in adipose tissue [27]. However, Hall and Guo claimed that low-fat diets increase both energy expenditure (26 kcal/day; *P* < 0.0001) and fat loss (16 g/day; *P* < 0.0001) [28], that can effectively decrease weight gain in long term which partly justifies our results.

In our study, replacing all three types of fatty acids (SFA, MUFA and PUFA) with carbohydrate contributed to the prevention of weight gain; however, in another cohort study, increasing the consumption of PUFAs and plant-based MUFA at the expense of carbohydrates was associated with less weight gain; and increasing consumption of SFA and trans-fat was associated with greater weight gain [29]. Undoubtedly, the intakes of different types of fatty acids and its major food sources are different in societies, and this difference can justify these conflicting results.

The effect of iso-caloric substitution of dietary macronutrients on weight or waist change is different by sex, which may be because weight change in women is more dependent on calorie consumption, insulin sensitivity [15] and hormone levels [30].

The prospective design of the study, using a valid and reliable FFQ, and analyzing data using iso-caloric substitution models are strengths of the present study. In the substitution model, the effect of substituting one nutrient intake with another was investigated while energy intake in the diet was kept constant [31]. Weight changes occur gradually at the population level, therefore our 3.6 year assessment period is consistent with the time course of weight change in response to a change in diet [32].

Some limitations should also be mentioned; first, despite using a validated FFQ in this study, FFQs, like other tools of dietary assessments, are subject to measurement errors. Second, the study participants are not representative of the whole Iranian population, which limits the generalizability of our findings to other Iranian and non-Iranian populations that have different proportions of macronutrients in their habitual diet compared to our study participants; third, the use of a substitution model is justified when studying the health effects of different macronutrients in iso-energetic conditions, this approach is only a mathematical model for dietary intakes and not a real-life condition. Finally, we did not have enough information about family income; therefore, in our study, it was not possible to investigate the effect of family income on macronutrient intakes.

## Conclusions

our findings demonstrated that the proportions of macronutrient intakes in habitual diets may be associated with obesity indices; higher proportions of plant protein in diet was associated with lower increase in BMI and WC, and decreased the risk of obesity during 3.6 years of follow-up, especially in men.

## Data Availability

The datasets generated and/or analysed during the current study are not publicly available due [REASON WHY DATA ARE NOT PUBLIC] but are available from the corresponding author on reasonable request.

## Abbreviations

BMI: Basal Metabolic Rate
EER: Estimated Energy requirement
EI: Energy intake
FCT: Food Composition Table
FFQ: Food frequency questionnaire
MAQ: Modifiable Activity Questionnaire
MUFAs: Monounsaturated Fatty Acids
PUFAs: Polyunsaturated Fatty Acids
SFAs: Saturated Fatty Acids
TLGS: Tehran Lipid and Glucose Study ()
USDA: United States Department of Agriculture
WC: Waist circumference

## References

[1] Budny A, Grochowski C, Kozlowski P, Kolak A, Kaminska M, Budny B (2019). Obesity as a tumour development triggering factor. Ann Agric Environ Med.

[2] Barzin M, Valizadeh M, Serahati S, Mahdavi M, Azizi F, Hosseinpanah F (2018). Overweight and obesity: findings from 20 years of the Tehran lipid and glucose study. Int J Endocrinol Metab.

[3] Romieu I, Dossus L, Barquera S, Blottiere HM, Franks PW, Gunter M (2017). Energy balance and obesity: what are the main drivers?. Cancer Causes Control.

[4] Vergnaud AC, Norat T, Mouw T, Romaguera D, May AM, Bueno-de-Mesquita HB (2013). Macronutrient composition of the diet and prospective weight change in participants of the EPIC-PANACEA study. PLoS One.

[5] Yancy WS, Olsen MK, Guyton JR, Bakst RP, Westman EC (2004). A low-carbohydrate, ketogenic diet versus a low-fat diet to treat obesity and hyperlipidemia: a randomized, controlled trial. Ann Intern Med.

[6] Brinkworth GD, Noakes M, Buckley JD, Keogh JB, Clifton PM (2009). Long-term effects of a very-low-carbohydrate weight loss diet compared with an isocaloric low-fat diet after 12 mo. Am J Clin Nutr.

[7] Bazzano LA, Hu T, Reynolds K, Yao L, Bunol C, Liu Y (2014). Effects of low-carbohydrate and low-fat diets: a randomized trial. Ann Intern Med.

[8] Bray GA, Smith SR, de Jonge L, Xie H, Rood J, Martin CK (2012). Effect of dietary protein content on weight gain, energy expenditure, and body composition during overeating: a randomized controlled trial. Jama.

[9] Lin Y, Bolca S, Vandevijvere S, De Vriese S, Mouratidou T, De Neve M (2011). Plant and animal protein intake and its association with overweight and obesity among the Belgian population. Br J Nutr.

[10] Azizi F, Ghanbarian A, Momenan AA, Hadaegh F, Mirmiran P, Hedayati M (2009). Prevention of non-communicable disease in a population in nutrition transition: Tehran lipid and glucose study phase II. Trials.

[11] Azizi F, Rahmani M, Emami H, Mirmiran P, Hajipour R, Madjid M (2002). Cardiovascular risk factors in an Iranian urban population: Tehran lipid and glucose study (phase 1). Soz Praventivmed.

[12] Asghari G, Rezazadeh A, Hosseini-Esfahani F, Mehrabi Y, Mirmiran P, Azizi F (2012). Reliability, comparative validity and stability of dietary patterns derived from an FFQ in the Tehran lipid and glucose study. Br J Nutr.

[13] Esfahani FH, Asghari G, Mirmiran P, Azizi F (2010). Reproducibility and relative validity of food group intake in a food frequency questionnaire developed for the Tehran lipid and glucose study. J Epidemiol.

[14] United States Department of Agriculture. Food composition table. Washington DC: USDA. [7 Jan 2015.]. Available from: http://www.nal.usda.gov/fnic/foodcomp.

[15] Azar M, Sarkisian E (1980). Food composition table of Iran.

[16] Ainsworth BE, Haskell WL, Whitt MC, Irwin ML, Swartz AM, Strath SJ (2000). Compendium of physical activities: an update of activity codes and MET intensities. Med Sci Sports Exerc.

[17] Kriska AM, Knowler WC, LaPorte RE, Drash AL, Wing RR, Blair SN (1990). Development of questionnaire to examine relationship of physical activity and diabetes in Pima Indians. Diabetes Care.

[18] Momenan AA, Delshad M, Sarbazi N, Rezaei Ghaleh N, Ghanbarian A, Azizi F (2012). Reliability and validity of the Modifiable Activity Questionnaire (MAQ) in an Iranian urban adult population. Arch Iran Med.

[19] US Department of Health and Human Services C, General fDCaP, http:// PADbLoI, www.cdc.gov/nccdphp/dnpa/physical/pdf/PA_Intensity_,. doi.

[20] Deibert P, Konig D, Schmidt-Trucksaess A, Zaenker KS, Frey I, Landmann U (2004). Weight loss without losing muscle mass in pre-obese and obese subjects induced by a high-soy-protein diet. Int J Obes Relat Metab Disord.

[21] Park Ki-Byeong, Park Hyun, Kang Jae-Heon, Kim Kyoungwoo, Cho Young, Jang Jinyoung (2018). Animal and Plant Protein Intake and Body Mass Index and Waist Circumference in a Korean Elderly Population. Nutrients.

[22] Layman DK, Evans EM, Erickson D, Seyler J, Weber J, Bagshaw D (2009). A moderate-protein diet produces sustained weight loss and long-term changes in body composition and blood lipids in obese adults. J Nutr.

[23] Baer DJ, Rumpler WV, Miles CW, Fahey GC (1997). Dietary fiber decreases the metabolizable energy content and nutrient digestibility of mixed diets fed to humans. J Nutr.

[24] Stenholm S, Harris TB, Rantanen T, Visser M, Kritchevsky SB, Ferrucci L (2008). Sarcopenic obesity: definition, cause and consequences. Curr Opin Clin Nutr Metab Care.

[25] Thompson Henry, McGinley John, Neil Elizabeth, Brick Mark (2017). Beneficial Effects of Common Bean on Adiposity and Lipid Metabolism. Nutrients.

[26] Samaha FF, Iqbal N, Seshadri P, Chicano KL, Daily DA, McGrory J (2003). A low-carbohydrate as compared with a low-fat diet in severe obesity. N Engl J Med.

[27] Hall KD (2018). A review of the carbohydrate-insulin model of obesity. Eur J Clin Nutr.

[28] Hall KD, Guo J (2017). Obesity Energetics: Body Weight Regulation and the Effects of Diet Composition. Gastroenterology.

[29] Liu X, Li Y, Tobias DK, Wang DD, Manson JE, Willett WC (2018). Changes in types of dietary fats influence long-term weight change in US women and men. J Nutr.

[30] Brown LM, Clegg DJ (2010). Central effects of estradiol in the regulation of food intake, body weight, and adiposity. J Steroid Biochem Mol Biol.

[31] Song M, Giovannucci E (2018). Substitution analysis in nutritional epidemiology: proceed with caution. Eur J Epidemiol.

[32] Hall KD, Sacks G, Chandramohan D, Chow CC, Wang YC, Gortmaker SL (2011). Quantification of the effect of energy imbalance on bodyweight. Lancet.

